# *Lactobacillus plantarum* producing a *Chlamydia trachomatis* antigen induces a specific IgA response after mucosal booster immunization

**DOI:** 10.1371/journal.pone.0176401

**Published:** 2017-05-03

**Authors:** Katarzyna Kuczkowska, Ine Myrbråten, Lise Øverland, Vincent G. H. Eijsink, Frank Follmann, Geir Mathiesen, Jes Dietrich

**Affiliations:** 1 Department of Chemistry, Biotechnology and Food Science, Norwegian University of Life Sciences (NMBU), 1432 Ås, Norway; 2 Statens Serum Institut, Department of Infectious Disease Immunology, Copenhagen, Denmark; University of California, San Francisco, Universit of California, Berkeley and the Childrens Hospital Oakland Research Institute, UNITED STATES

## Abstract

Mucosal immunity is important for the protection against a wide variety of pathogens. Traditional vaccines administered via parenteral routes induce strong systemic immunity, but they often fail to generate mucosal IgA. In contrast, bacteria-based vaccines comprise an appealing strategy for antigen delivery to mucosal sites. Vaginal infection with *Chlamydia trachomatis* can develop into upper genital tract infections that can lead to infertility. Therefore, the development of an effective vaccine against Chlamydia is a high priority. In the present study, we have explored the use of a common lactic acid bacterium, *Lactobacillus plantarum*, as a vector for delivery of a *C*. *trachomatis* antigen to mucosal sites. The antigen, referred as Hirep2 (H2), was anchored to the surface of *L*. *plantarum* cells using an N-terminal lipoprotein anchor. After characterization, the constructed strain was used as an immunogenic agent in mice. We explored a heterologous prime-boost strategy, consisting of subcutaneous priming with soluble H2 antigen co-administered with CAF01 adjuvant, followed by an intranasal boost with H2-displaying *L*. *plantarum*. The results show that, when used as a booster, the recombinant *L*. *plantarum* strain was able to evoke cellular responses. Most importantly, booster immunization with the *Lactobacillus*-based vaccine induced generation of antigen-specific IgA in the vaginal cavity.

## Introduction

Mucosal immunization is currently receiving much attention in vaccinology. Most pathogens enter the body through mucosal surfaces and the development of effective vaccines able to inhibit invasion at the mucosal surfaces is needed. Mucosal immunization targets the local mucosa-associated lymphoid tissue (MALT) inducing a mucosal memory response, where tissue-resident memory T and B cells play a key role in generating local protection against invading pathogens [1]. Traditional vaccination via parenteral routes, such as intramuscular or subcutaneous routes, usually produces no or very weak mucosal immunity [2]. In contrast, administration of vaccines through mucosal routes, e.g. nasal or oral, may induce immune responses in both mucosal and systemic compartments [2, 3].

One of the most intensively studied strategies for vaccine delivery to mucosal sites entails the use of live microorganisms, including both attenuated pathogens and commensals. The use of bacteria as vaccine delivery vectors is highly advantageous due to several aspects, such as easy production and manipulation, low-cost manufacturing and safe disposal. Attenuated pathogens are attractive, since they can induce immunity against heterologous antigens, as well as promote protection against the pathogen itself [4]. However, unstable phenotypes and the risk of reversion to virulence make the use of attenuated pathogens challenging and risky. In this regard, non-pathogenic food-grade bacteria, particularly lactic acid bacteria (LAB), are a promising alternative to attenuated pathogens [5].

The utilization of LAB as a carrier for prophylactic and therapeutic molecules has been studied for more than 25 years and this includes studies on developing LAB for vaccine delivery to mucosal sites [5–10]. LAB offer several advantages, such as easy cultivation and access to a versatile genetic engineering toolbox. Moreover, LAB can adhere to epithelial cells and thus allow for longer persistence of delivered biomolecules at mucosal sites. Additionally, several LAB species can modulate the immune system of the host [11–14]. Immunomodulatory properties are particularly common for members of the genus *Lactobacillus*, however, the nature of immunomodulation varies between strains [15–17]. *Lactobacillus plantarum* was characterized in terms of its adjuvant properties several decades ago, when the bacterium was identified as a major adjuvant in a mistletoe preparation [18, 19]. Accumulating data [11–17] indicate that *L*. *plantarum* is an appropriate candidate for antigen delivery to mucosal sites. As an example, it has been shown that intranasal or oral immunization with *L*. *plantarum* producing tetanus toxin induced protection in mice [20–22].

*Chlamydia trachomatis* is a causative agent of genital and ocular disease. The sexually transmitted infection remains the most commonly reported infectious disease in many countries and the infection rate is increasing each year [23, 24]. Due to the lack of symptoms (75 – 90% cases), the majority of *C*. *trachomatis* infections remains undiagnosed, which hampers control and intervention [24]. Moreover, although a diagnosed *C*. *trachomatis* infection can be easily treated by antibiotic therapy, increased susceptibility for re-infection has been reported for cured patients [25, 26]. Therefore, the development of an efficient prophylactic vaccine is needed to reduce incidences of infection and to protect humans from a harmful disease. One of the most intensively studied antigens for vaccine development is the major outer membrane protein (MOMP) of *C*. *trachomatis*, which is an immunodominant antigen, eliciting both humoral and cellular immune responses [27].

In the present study, we have engineered a *L*. *plantarum* strain producing a *C*. *trachomatis* antigen and studied its potential as mucosal booster vaccine against *C*. *trachomatis* genital infection. We utilized a multivalent antigen based on MOMP variable domain 4 (VD4) from different *C*. *trachomatis* serovars (serovars D, E, F and G; recognized as the most prevalent serovars of *C*. *trachomatis* causing genital infection), known as heterologous immuno-repeat 2 (Hirep2) [28]. The Hirep2 antigen (referred to as H2) was N-terminally fused to a lipoprotein anchor called Lp_1261, to direct the fusion protein to the surface of *L*. *plantarum*. The constructed strain, hereafter referred to as *Lp*_H2, was evaluated in a heterologous prime-boost vaccination regimen, i.e. as a booster vaccine. We chose this vaccine strategy as it was recently shown that administering a mucosal booster vaccine on top of systemic immunity is particularly effective in terms of achieving mucosal immunity [29]. Mice were primarily immunized with H2 emulsified in cationic adjuvant formulation 1 (CAF01), referred as H2-CAF01, via the subcutaneous route, followed by intranasal booster vaccination with *Lp*_H2. We investigated the potential of the *L*. *plantarum*-based vaccine in terms of its ability to evoke cellular and humoral systemic immune responses, as well as induction of an IgA antibody response at mucosal sites.

## Materials and methods

### Ethics statement

Experiments were conducted in accordance with a local animal protection committee by Danish Animal Experiments Inspectorate Permit 2004-561-868 (of January 7, 2004) and with the regulations of the Danish Ministry of Justice and animal protection committees, and in compliance with the EU Directive 2010/63/EU. The experiments were approved by the Statens Serum Institut IACUC headed by DVM Kristin E. Engelhart Illigen.

### Animal handling

Female 6–8 weeks old B6C3F1 mice were purchased from Envigo Laboratories (The Netherlands) and randomly assigned to cages at the animal facility at Statens Serum Institut upon arrival. Animals were housed under standard environmental conditions and provided standard food and water ad libitum. All mice were euthanized by CO_2_/O_2_ exposure. All procedures have been refined to provide for maximum comfort and minimal stress to the animals.

### Bacterial strains, plasmids and growth conditions

The bacterial strains and plasmids used in this study are listed in Table 1. *Lactobacillus plantarum* strains were cultured in MRS broth (Oxoid Ltd., Basingstoke, United Kingdom) at 37°C without shaking. *Escherichia coli* TOP10 cells (Invitrogen) were grown in Brain Heart Infusion (BHI, Oxoid) broth at 37°C with shaking. Erythromycin was added to a final concentration of 10 μg/mL for *L*. *plantarum* or 200 μg/mL for *E*. *coli*. For plates, liquid medium was solidified by adding 1.5% (w/v) agar.

**Table 1:** Plasmids and strains used in this study.

Strain or plasmid	Description	Reference
**Plasmids**		
pUC-Hirep2-DC	Amp^r^; pUC57 vector with synthetic *hirep2dc* gene fragment	GenScript
pEv	Ery^r^; control plasmid (“empty vector”)	[30]
pLp_1261Inv	Ery^r^; pLp_2588AmyA [31] derivative, encoding a lipoprotein anchor sequence from Lp_1261 fused to an *inv* gene fragment	[30]
pLp_1261H2-DC	Ery^r^, pLp_1261Inv derivative, where the *inv* gene fragment is replaced by a gene fragment encoding Hirep2-DC	This study
**Strains**		
*L*. *plantarum* WCFS1	Host strain	[32]
*E*. *coli* TOP10	Host strain	Invitrogen
*Lp*_H2	*L*. *plantarum* WCFS1 harboring pLp_1261H2-DC; for surface display of the H2 antigen using an N-terminal lipoprotein anchor	This study
*Lp*_Ev	*L*. *plantarum* WCFS1 carrying pEv (empty vector); used as a negative control strain	[30]

### DNA manipulations and plasmid construction

**Fig 1:**
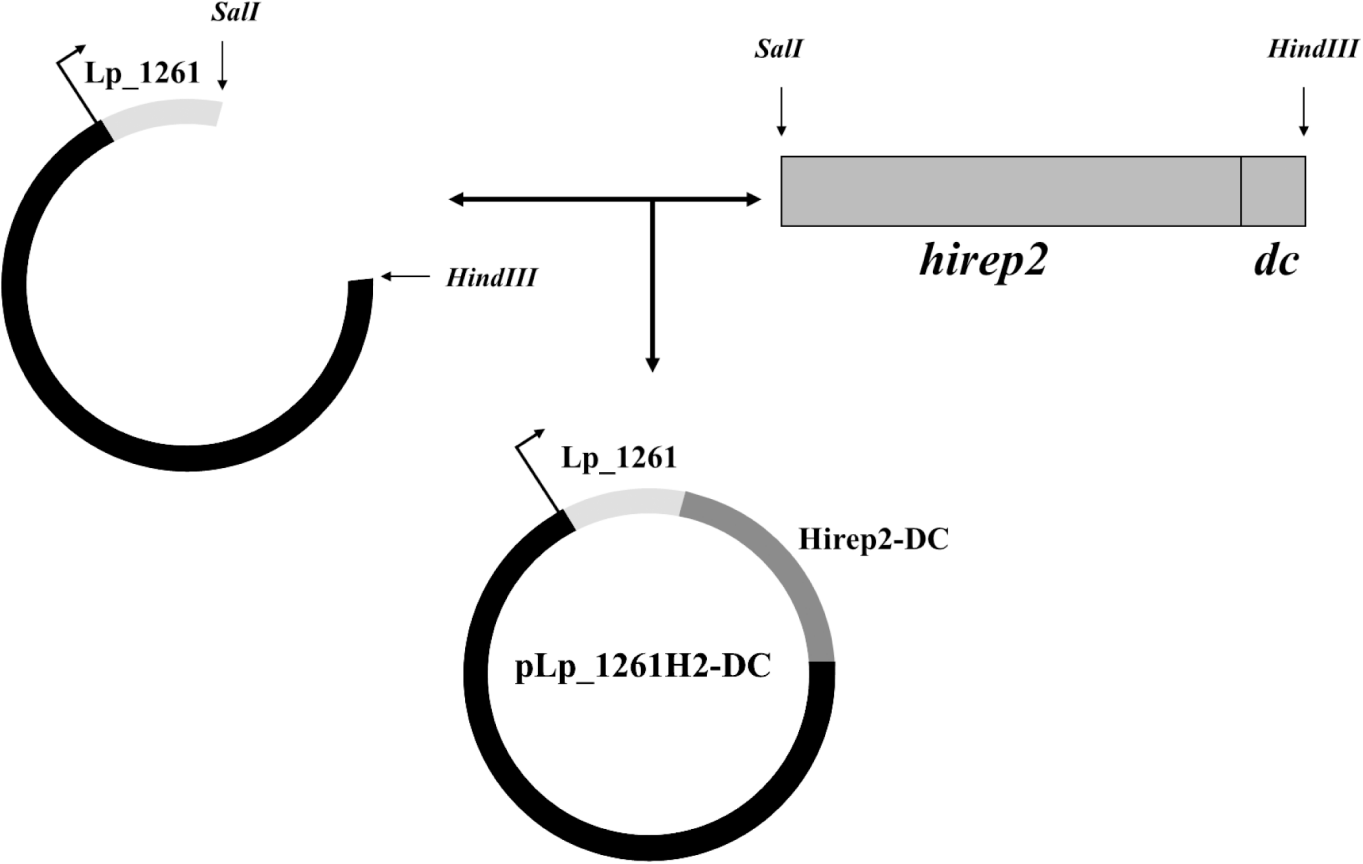
Construction of the expression vector for production of the H2 antigen fused to the Lp_1261 lipoprotein anchor. *SalI* and *HindIII* restriction sites enabled easy gene exchange. The gene fragment encoding the Hirep2-DC antigen (darker gray) was introduced into a plasmid harboring the lipoprotein anchor derived from *L*. *plantarum* protein Lp_1261 (light gray). The complete gene construct was translationally fused to the inducible promoter that is indicated by the thin arrow.

The basic outline of the constructed expression vector is shown in Fig 1. Plasmid pLp_1261Inv [30], developed for inducible expression and anchoring, via N-terminal lipoprotein anchor, of invasin from *Yersinia pseudotuberculosis* was used as a starting point. A *hirep2dc* gene fragment was designed such as to fuse a 12-residue long sequence (FYPSYHSTPQRP) binding dendritic cells (DCs) [33, 34] to the C-terminal end of Hirep2. Restriction sites, *SalI* and *HindIII*, were introduced to facilitate further manipulations. The *hirep2dc* DNA fragment, encoding the Hirep2 antigen fused to DC-targeting sequence (Hirep2-DC) was codon optimized for expression in *L*. *plantarum*, synthesized at Genscript (Piscataway, NJ, USA), and cloned into a pUC57 plasmid, yielding pUC-Hirep2-DC. The pUC-Hirep2-DC plasmid was digested with *SalI* and *HindIII*. The resulting 873 bp Hirep2-DC-encoding fragment was cloned into *SalI/HindIII* digested pLp_1261Inv, yielding pLp_1261H2-DC (Fig 1).

The pLp_1261H2-DC plasmid was first transformed into *E*. *coli* TOP10. Positive clones were screened by PCR and restriction enzyme digestion after which the PCR-amplified fragments were verified by sequencing using primers SekF (GGCTTTTATAATATGAGATAATGCCGAC) and SekR (CCTTATGGGATTTATCTTCCTTATTCTC). The plasmid was purified using the PureYield^TM^ Plasmid Miniprep System (Promega Corporation, Madison, WI, USA) and electroporated into *L*. *plantarum* cells according to Aukrust *et al*.[35].

### Purification of recombinant Hirep2 and Hirep1

The Hirep1 protein is based on MOMP VD4 from *C*. *trachomatis* serovars D, E and F, while Hirep2 consists of Hirep1 and an additional VD4 from serovar G of *C*. *trachomatis* [28]. Based on the Hirep2/Hirep1 amino acid sequences [28] with an added N-terminal histidine tag, synthetic DNA constructs, codon optimized for expression in *E*. *coli*, were prepared, followed by insertion into the pJexpress 411 expression vector (DNA2.0, Newark, CA, USA). Purification was done essentially as described elsewhere [36].

### Western blot analysis

To analyze expression of Lp_1261H2-DC in *L*. *plantarum*, cells from a 50 mL culture were harvested 3 h after induction, cell-free protein extracts were prepared, and proteins were separated by SDS-PAGE and transferred to a nitrocellulose membrane, as described elsewhere [37]. The membrane was blocked by soaking in 5% (w/v) dry milk (Difco) in Tris Buffer Saline (TBS, 150 mM NaCl, 10 mM Tris-HCl; pH 7.4) supplemented with 0.1% (v/v) Tween-20 (Sigma-Aldrich) for 1 h at room temperature, followed by incubation with the anti-Hirep2 (anti-H2) primary antibody (produced in rabbit) in TBS with 3% (w/v) bovine serum albumin (BSA, Sigma-Aldrich), at 4°C, overnight. Subsequently, the blot was incubated with polyclonal HRP-conjugated goat anti-rabbit IgG (Invitrogen) diluted 1:5 000 in TBS containing 0.1% (v/v) Tween-20 and 5% (w/v) dry milk, for 1 h, at room temperature. Proteins were visualized using the SuperSignal™ West Pico Chemiluminescent Substrate (Termo Scientific) and signals were documented using an Azure c400 system and AzureSpot Analysis Software (Azure Biosystems, Dublin, CA, USA), following the manufacturer’s instructions.

### Semi-quantitative Western blot

To estimate the total amount of H2 antigen produced in *L*. *plantarum*, bacterial cell lysate was prepared. *L*. *plantarum* cells were harvested 3 h after induction, as described elsewhere [37]. Approximately 1 x 10^9^ CFU were treated with 10 mg/mL lysozyme (Sigma-Aldrich) and 15 U/mL mutanolysin (Sigma-Aldrich) in 125 μL Glucose-Tris Buffer (GTB, 50 mM glucose, 25 mM Tris-HCL pH 8.0, 10 mM EDTA pH 8.0) for 30 min at 37°C. Treated cells were transferred to -80°C, incubated for 15 min, and subsequently boiled for 15 min. A solution of the Hirep1 protein with known concentration was used to make a standard curve. We used Hirep1 recombinant protein instead of Hirep2 for practical reasons. Serial dilutions of the *L*. *plantarum* cell lysate were tested to find dilutions giving signals within the standard curve. The bacterial lysate was diluted in sterile distilled H_2_O in the following ratios: 1:20, 1:30, 1:40, 1:50. The *L*. *plantarum* lysate dilutions and dilutions of the standard protein were subjected to SDS-polyacrylamide gel electrophoresis and the proteins were then transferred to a nitrocellulose membrane as described above. The proteins were detected using the SNAP i.d.® 2.0 Protein Detection System (Merck kGaA Darmstadt, Germany) using a rabbit anti-H2 primary antibody (which also binds to Hirep1) and HRP-conjugated goat anti-rabbit IgG as the secondary antibody, and visualized (as above). AzureSpot Analysis Software was used to determine the total quantity of H2, using the standard curve. The concentration was calculated for each dilution of the cell lysate containing H2 and the average of all dilutions was used as the estimated concentration.

### Flow cytometry and indirect immunofluorescence microscopy of *L*. *plantarum* expressing H2

Approximately 1 x 10^9^ CFU of induced bacterial cultures were resuspended in 50 μL PBS containing 1% (w/v) BSA and 5 μg/mL anti-H2, followed by incubation for 30 min at 37°C. Cells were centrifuged at 5 000 × *g* for 5 min and washed 3 times with PBS/1% BSA. Subsequently, cells were resuspended in 50 μL PBS/1% BSA and 0.8 μL FITC-conjugated anti-rabbit IgG secondary antibody (Sigma-Aldrich), followed by incubation in darkness and at room temperature for 30 min. Cells were collected by centrifugation, washed 3 times with PBS/1% BSA and resuspended in 100 μL PBS without BSA. The bacterial suspensions were immediately analyzed by flow cytometry using a MACSQuant analyzer (Miltenyi Biotec GmbH, Bergisch Gladbach, Germany), following the manufacturer’s instructions. For indirect immunofluorescence microscopy, bacteria were visualized under a Zeiss Axio Observer.z1 microscope (Zeiss, Germany) and the fluorescence was acquired by EX 450–490 nm and EM 500–590 nm (EX, excitation; EM, emission) at magnification x100.

### Immunization protocol

Mice were divided in 4 groups (*n* = 8). The experimental groups and the immunization regimen are described in Table 2. 5 μg of soluble H2 antigen was emulsified in 200 μL of cationic adjuvant formulation 1 (CAF01) [38] and so made H2-CAF01 vaccine was administered by the subcutaneous route. *L*. *plantarum* cells were prepared as described elsewhere [37]. For practical reasons, bacterial cultures were harvested 3–4 days before immunization and the live cells were stored as cell pellets, at 4°C. At the day of administration, three weeks after the H2-CAF01 immunization, the bacterial pellets were resuspended in PBS and given intranasally to anesthetized mice. The immunization dose contained approximately 1 x 10^9^ CFU of *L*. *plantarum* in 20 μL PBS and two such doses were given on two consecutive days, according to well-established protocols described in the literature, e.g. [21, 39, 40] and to the pilot experiment performed prior to the present experiment (data not shown). Non-immunized (Naïve) and non-boosted (H2) mice receiving only H2-CAF01 subcutaneously were used as controls. A group of mice that received only the bacterial-based intranasal immunization without subcutaneous priming was also included. All mice were euthanized 4 weeks after the last immunization. Blood, spleens, lungs and vaginal washes were collected and analyzed. The cellular antigen specific immune response was examined in splenocytes and PBMC by measuring IFN-γ in cell supernatants of H2-stimulated cells. Furthermore, the antibody responses in blood (IgG and IgA levels in plasma), antigen-specific IgA production at mucosal sites (vaginal washes and lungs) and the recognition of a neutralizing epitope by IgA from vaginal washes and lungs were investigated.

**Table 2:** Experimental groups and immunization protocol.

Mouse group	Immunization 1	Immunization 2	Immunization 3
	Day 1	Day 22	Day 23
Naïve	-	-	-
H2	H2-CAF01,	-	-
	200 μL, Sc*		
H2/Lp_H2	H2-CAF01,	*Lp_*H2	*Lp*_H2
	200 μL, Sc*	20 μL, IN**	20 μL, IN**
Lp_H2	-	*Lp*_H2	*Lp_*H2
		20 μL, IN**	20 μL, IN**

*Sc–subcutaneous immunization

**IN–intranasal immunization

### Sample collection and analysis

#### Isolation of plasma and PBMCs from blood

Blood was collected into EDTA-treated tubes and centrifuged at 1 350 × *g* to separate plasma. Murine PBMCs were purified from blood in EDTA tubes using a density gradient. After washing, freshly isolated cells were used in cellular assays (described below).

#### Isolation of splenocytes

Spleens were collected, mashed through 100 μm Corning® cell strainers (Sigma-Aldrich), centrifuged at 300 × *g* for 10 min at room temperature and washed twice with RPMI 1640 medium (Gibco Invitrogen).

#### Vaginal washes

Vaginal samples were collected from individual mice by flushing the vagina with 100 μL of sterile PBS. Next, vaginal washes were treated with Bromelain (Sigma); samples were diluted 1:5 with PBS/1% BSA and incubated for 1 h at 37°C to solubilize the mucus. Samples were stored at -80°C until analysis.

#### Lung samples

Lungs were collected from individual mice and transferred to gentleMACS C Tubes (Miltenyi Biotec GmbH, Bergisch Gladbach, Germany) containing 2mL RPMI 1640 supplemented with 5% fetal calf serum (FCS), and 0.8mg/mL Collagenase type IV (Sigma-Adrich, St. Louis, MO) and dissociated into 1–2mm pieces using the gentleMACS Octo dissociator (Miltenyi Biotec GmbH), followed by 1h incubation at 37°C. The samples were returned to the gentleMACS and dissociated, followed by centrifugation (700 × *g*, 5min). Supernatants were collected for antibody detection and stored at -80°C until further analysis.

### Cellular assay for splenocytes and PBMCs

2 x 10^5^ of freshly isolated cells were seeded in round bottom 96-well microtiter plates (Nunc) in 200 μL RPMI 1640 supplemented with 50 μM 2-mercaptoethanol, 1 mM glutamine, 1% pyruvate, 1% penicillin-streptomycin, 1% HEPES and 10% FCS (Gibco, Invitrogen). The cells were stimulated with 5 μg/mL of soluble H2 antigen in triplicates. Cells were maintained in a humidified incubator at 37°C and 5% CO_2_. Non-stimulated cells were included as a negative control. Supernatants were harvested after 72 h of stimulation and stored at -20°C until further analysis.

### ELISA (Enzyme-linked immunosorbent assay)

MaxiSorp ELISA plates were coated with antigen/antibody in carbonate buffer (SSI Diagnostics, Copenhagen, Denmark) overnight at 4°C and then blocked with 2% BSA (w/v) in PBS for at least 1.5 h at room temperature. The samples were applied to coated and washed plates and incubated for 2 h at room temperature. Subsequently, plates were incubated with HRP-conjugated specific antibody diluted in PBS/1% BSA for 2 h. TMS Ready-to-use substrate (Kem-En-Tec Nordic A/S, Taastrup, Denmark) was used for color development and the color reaction was stopped by 0.2 M sulfuric acid. The OD at 450 nm with the reference wavelength set to 650 nm was measured using a Sunrise Plate Reader (Tecan Group Ltd., Männedorf, Switzerland).

### Antigen-specific IgG and IgA in plasma and IgA in mucosal washes

ELISA plates were coated with 1 μg/mL of H2 antigen. 10-fold dilutions of plasma samples and 5-fold dilutions of vaginal and lung samples were applied. HRP-conjugated anti-mouse IgA antibody (AH Diagnostics, Aarhus, Denmark) diluted 1:5 000 or HRP-conjugated anti-mouse IgG antibody (AH Diagnostics) diluted 1:20 000 were used for detection of IgA or IgG, respectively.

### Recognition of a neutralizing epitope

ELISA plates were coated with 10 μg/ml of P30 peptide (containing the neutralizing epitope sequence LNPTIAG; [28]) or with H2, as a control. 5-fold dilutions of vaginal washes and lung samples were applied in triplicates. HRP-conjugated anti-mouse IgA antibody (AH Diagnostics, Aarhus, Denmark) diluted 1:5 000 was used for detection of IgA recognizing the neutralizing epitope.

### IFN-γ production

ELISA plates were coated with purified rat anti-mouse IFN-γ antibody (BD Pharmingen, San Diego, CA, USA). The supernatants harvested from cellular assays were applied in triplicates. Subsequently, the plates were incubated with biotin-labeled rat anti-mouse IFN-γ (BD Pharmingen) as a capture antibody and HRP-conjugated streptavidin (Zymed, San Francisco, CA, USA) was used for detection.

### Statistical tests

Statistical significance was determined using one-way ANOVA with Tukey's post hoc test for the analysis of IFN-γ production by splenocytes and in the analysis of the recognition of the neutralizing epitope. One-way ANOVA with Dunnett's post hoc test was used for analysis of IgA levels at the lowest dilutions. The software used was GraphPad Prism (GraphPad Software, San Diego, CA, USA). Results are presented as a mean ± SEM. Statistical significance is shown as follows: *, p < 0.05; **, p < 0.01.

## Results

### Construction of *L*. *plantarum* for surface-display of the H2 antigen

The expression vector was generated as described in the Material and Methods section and outlined in Fig 1. We used an N-terminal lipoprotein anchor derived from the Lp_1261 protein, which ensures covalent binding to the cell membrane [30]. The N-terminal end of Hirep2 was translationally fused to the anchor sequence using an engineered *Sal*I restriction site. A DC-targeting peptide was fused to the C-terminal end of the antigen. Considering the N-terminal anchoring mode, in this set up, the DC-binding peptide was expected to be the most external part of the anchored protein, likely protruding from the bacterial cell surface. In some experiments, we used a *L*. *plantarum* strain harboring the empty vector, pEv [30], as a negative control.

### Characteristics of *L*. *plantarum* displaying H2 on its surface

**Fig 2:**
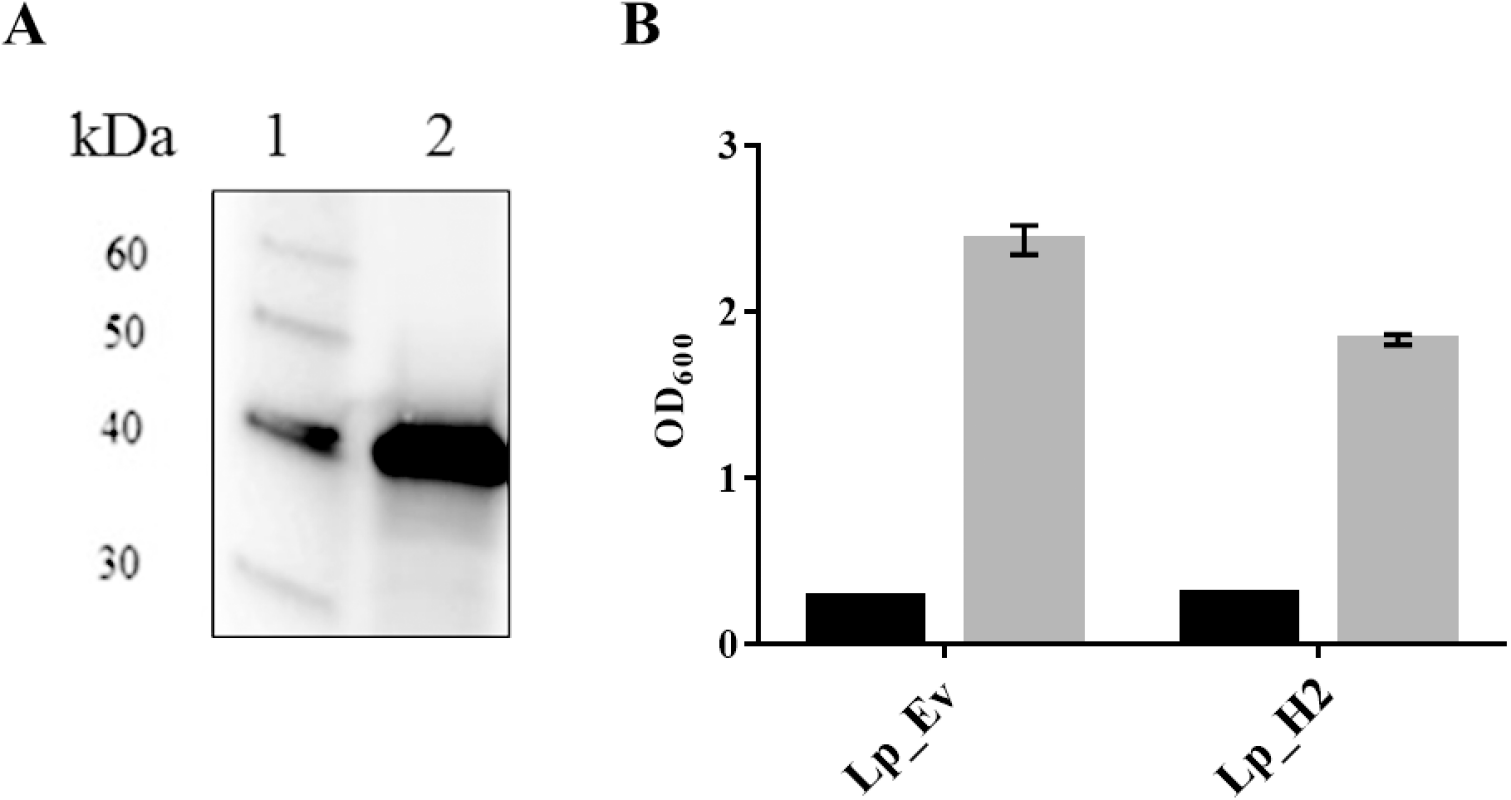
**Detection of anchor-fused H2 antigen (A) and cell growth of *L*. *plantarum* producing the H2 antigen (B).** Panel A shows Western blot analysis of a cell-free protein extract from *Lp*_H2 (lane 2) and a molecular mass standard (1). The predicted molecular mass of the Lp_1261H2-DC protein is 37 kDa. Panel B shows the growth rate of *Lp*_H2 and *Lp*_Ev, used as a control. The OD_600_ was measured at the induction point (black bars) and 3 h after induction (gray bars) for both strains. The data are presented as means of triplicates ± SEM.

To examine whether the recombinant antigen was produced in bacterial cells, we subjected cell-free crude protein extracts from induced cells to Western blotting (Fig 2A). The protein extract from the analyzed strain showed one distinct band of correct size (the expected molecular weight was 37 kDa), showing that the Lp_1261H2-DC hybrid protein was successfully produced in *L*. *plantarum*.

Expression and secretion of heterologous protein may be stressful for the growing cells. Therefore, we investigated whether production of lipoprotein-anchored H2 had a negative impact on the growth of *L*. *plantarum*. We observed only slightly reduced growth for *Lp*_H2 compared to *Lp*_Ev, a control strain, (Fig 2B), implying that production of the antigen had only limited effects on bacterial fitness.

**Fig 3:**
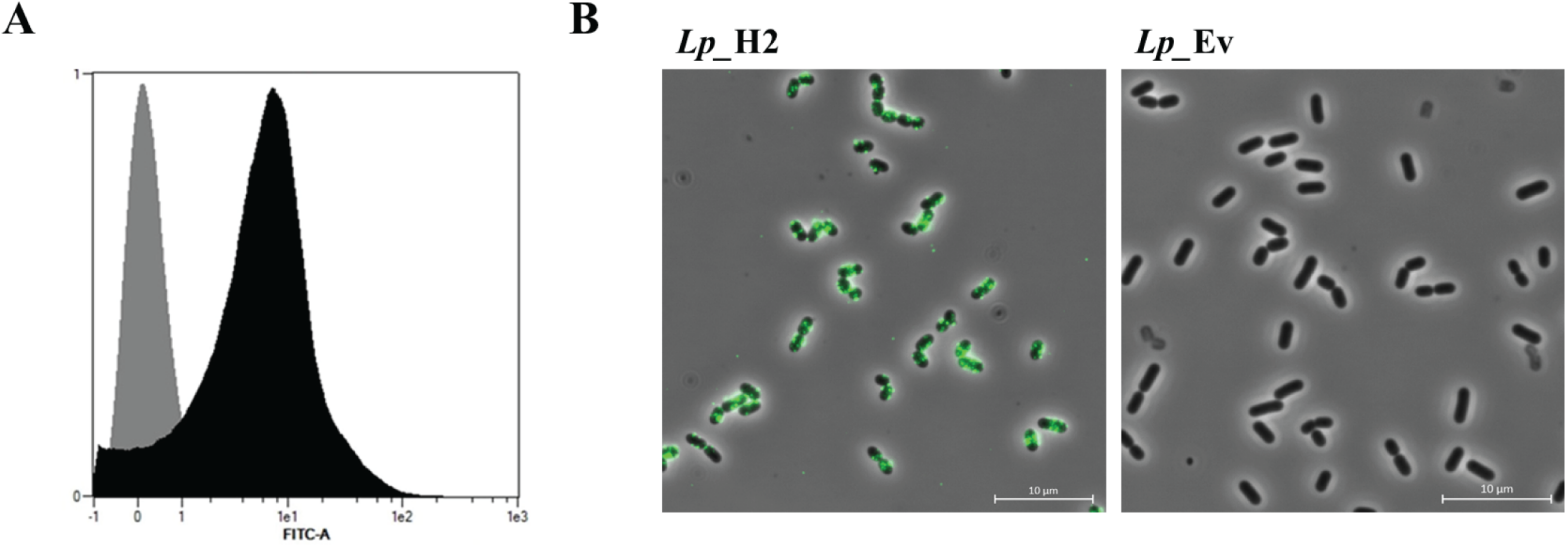
**Presence of the H2 antigen at the *L*. *plantarum* surface detected by flow cytometry (A) and fluorescence microscopy (B).** Cells were probed with an anti-H2 antibody. Panel A shows flow cytometry analysis for *Lp_*H2 (black histogram) and *Lp*_Ev used as a negative control (gray histogram). Panel B shows indirect immunofluorescence microscopy of the indicated strains. The data presented are from one representative experiment. Each experiment was performed three independent times, giving similar results.

In order to check the surface localization of the H2 antigen, bacterial cells of *Lp*_H2 and *Lp_*Ev (negative control) were labelled with anti-H2 antibody and examined by two methods: flow cytometry and fluorescent microscopy (Fig 3). Flow cytometry analysis (Fig 3A) showed increased fluorescence intensity for the H2-producing strain compared to the negative control strain, suggesting the presence of the antigen on the bacterial surface. These observations were confirmed by fluorescence microscopy (Fig 3B), which showed clear positive signals for cells expected to display the H2 antigen on the surface. Taken together, the findings from these experiments indicate that the H2 antigen was successfully displayed on the bacterial surface.

### Amount of antigen produced by the *Lp*_H2 strain

**Fig 4:**
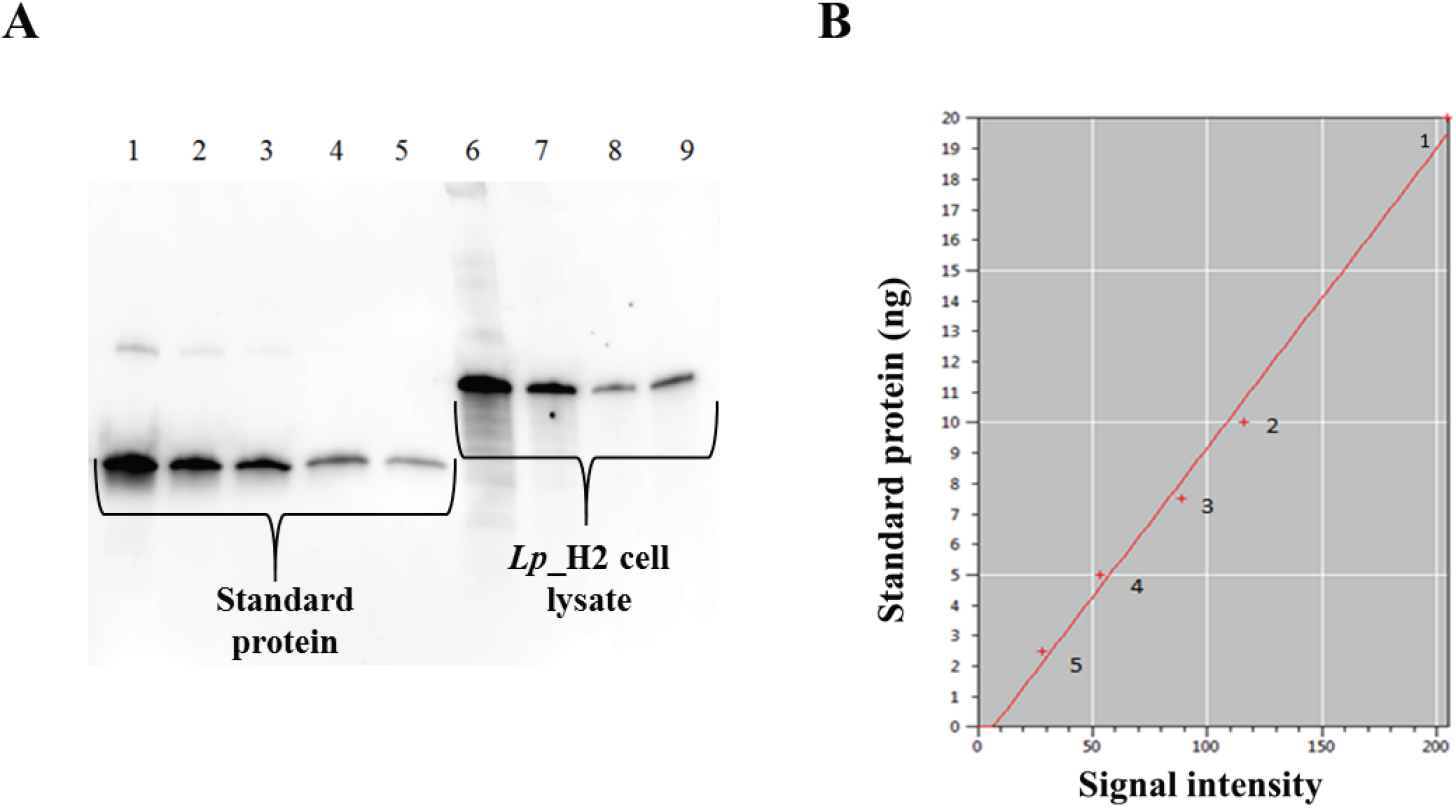
Estimation of the total amount of antigen by semi-quantitative Western blotting. Serial dilutions of bacterial cell lysate from 1 x 10^9^ CFU of induced *Lp_*H2 and dilutions of purified Hirep1 with known concentration were subjected to Western blotting and proteins were detected using the anti-H2 antibody (which also binds to Hirep1 protein). Panel A shows a typical Western blot: wells 1–5, standard protein (20 ng, 10 ng, 7.5 ng, 5 ng and 2.5 ng, respectively); wells 6–9, lysate of *Lp*_H2, diluted 20-fold, 30-fold, 40-fold and 50-fold, respectively. Signal intensities of different dilutions of the standard protein were used to make a standard curve that is presented in panel B. The marked points on the standard curve correspond with the amount of standard protein used: (1) - 20 ng, (2) - 10 ng, (3)– 7.5 ng, (4) - 5 ng, (5) - 2.5 ng.

A proper antigen concentration is essential for effective vaccination. Since the goal is to use the *L*. *plantarum* producing H2 antigen as a vaccine, we estimated the total amount of produced antigen in 1 x 10^9^ CFU of the *Lp*_H2 strain. To do so, we performed semi-quantitative Western blots using dilution series of bacterial cell lysate containing the antigen (Fig 4A). We employed a preparation of recombinantly produced, purified Hirep1 (a protein based on MOMP VD4 from *C*. *trachomatis* serovars D, E and F [28]) with known concentration to make a standard curve based on band intensities in the Western blot (Fig 4B). Using this approach, the total amount of H2 in one immunization dose consisting of 1 x 10^9^ CFU of *L*. *plantarum* was estimated to be approximately 7 μg, which is an acceptable antigen dose in mice [28].

### H2-specific IFN-γ and antibody responses and recognition of a neutralizing epitope by mucosal IgA

**Fig 5:**
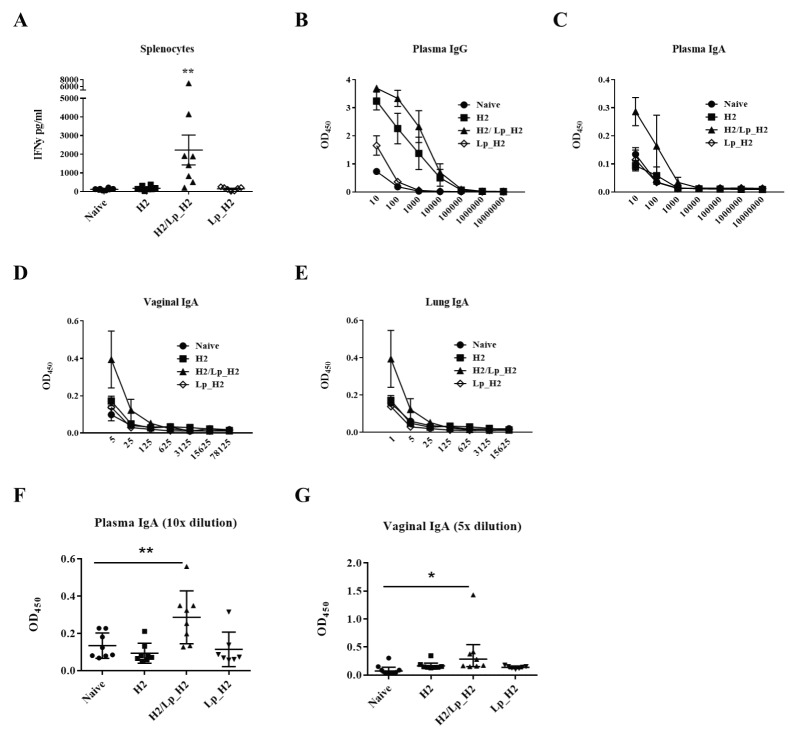
Immune responses induced by the booster *Lp_*H2 vaccine. Panel A presents the cellular response measured by H2-specific IFN-γ production in splenocytes. Cells were purified from individual mice and stimulated with H2 for 72 h. Produced IFN-γ was measured in harvested supernatants, by ELISA. Each point represents an individual mouse and the overall results per group are presented as a mean ± SEM (*n* = 8 for naive, H2 and H2/Lp_H2 groups, *n* = 7 for Lp_H2). Statistical significance was determined using one-way ANOVA with Tukey's post hoc test and is indicated as follows: **, p < 0.01. Panels B—E show antibody responses: H2-specific IgG in plasma (B) and H2-specific IgA in plasma (C), vaginal washes (D) and lung fluids (E). The samples from individual mice were serially diluted and added to H2-coated plates and specific IgG and IgA levels were measured by ELISA. The individual points represent the average OD_450_ values per group ± SEM (*n* = 8 for naive, H2 and H2/Lp_H2 groups, *n* = 7 for Lp_H2) at each dilution. Panels F and G show H2-specific IgA in 10-fold diluted plasma samples (F) and 5-fold diluted vaginal samples (G). Each point represents an individual mouse and the overall results per group are presented as a mean ± SEM (*n* = 8 for naive, H2 and H2/Lp_H2 groups, *n* = 7 for Lp_H2). Statistical significance was determined using one-way ANOVA with post hoc Dunnett's test and is shown as follows: *, p < 0.05; **, p < 0.01.

An immunization experiment with groups of eight mice was conducted as described in Materials and methods. The cellular assays showed that only the group that received the intranasal booster immunization with *Lp*_H2 exhibited significantly increased IFN-γ secretion in splenocytes stimulated with the soluble H2 antigen (Fig 5A). A similar trend was observed for PBMCs (data not shown). No increased IFN-γ production was observed for naïve mice, mice that only had received the parenteral immunization (H2) or mice that only had received the intranasal *Lp*_H2 vaccine (Lp_H2; see Table 2 for immunization protocol).

Measurements of the antigen-specific IgG levels in blood showed that subcutaneous immunization with H2-CAF01 resulted in high levels of H2-specific IgG in blood. Nonetheless, an intranasal boost with *Lp*_H2 slightly increased the IgG response (Fig 5B). We also observed slightly increased IgG levels in mice that had received *Lp*_H2 without priming (compared to non-vaccinated mice), but these levels were much lower than those obtained for the subcutaneously vaccinated groups (Fig 5B).

In order to determine whether the intranasal boost with *Lp*_H2 led to induction of mucosal immunity, we measured IgA levels in blood and at mucosal sites. We detected an elevated level of H2-specific IgA in plasma for the group receiving both the prime and booster vaccines (Fig 5C and 5F). Importantly, the IgA analysis in mucosal samples showed that intranasal boosting with the *Lactobacillus*-based vaccine generated H2-specific IgA in the vaginal washes (as well as in perfused lung) (Fig 5D, 5E and 5G). The elevation of IgA levels in plasma (at 10-fold dilution) and vaginal IgA (5-fold dilution) in H2/Lp_H2 mice compared to naïve mice was statistically significant (Fig 5F and 5G). The same trend was observed when testing recognition of a neutralizing epitope (containing the sequence LNPTIAG [28]) by IgA (S1 Fig).

Taken together, these observations show that subcutaneous priming with H2-CAF01 followed by intranasal boosting with *Lp*_H2 elicited increased H2-specific mucosal immunity.

## Discussion

In the present study, we examined the potential of *L*. *plantarum* as a carrier for a mucosal vaccine against *Chlamydia* infections. The bacterium was engineered to display a *C*. *trachomatis* antigen on its surface thought to be accessible for recognition by the immune system. We applied the engineered strain as a booster vaccine in a heterologous prime-boost immunization regimen. Several studies on *Chlamydia* vaccines have explored prime-boost regimes, showing protection against a genital *C*. *muridarum* challenge in mice [41], as well as generation of anti-chlamydia antibodies in the ocular lavage fluids [42]. In our study, the main goal was to investigate whether antigen-displaying *L*. *plantarum* administered to mucosal sites could elicit mucosal immune responses in mice with already established specific systemic immunity.

We used the recombinant H2 antigen since previous studies had shown that H2 induces protective levels of neutralizing antibody titers [28]. The H2 antigen constitutes the C-terminal part of CTH522 [28], a novel *Chlamydia* vaccine that is currently in clinical trials. H2-expressing *L*. *plantarum* strains could possibly find application as a booster vaccine given after CTH522 vaccination, to increase neutralizing antibody titers.

We used the Lp_1261 lipoprotein anchor for surface-display of the H2 antigen, because this anchor had previously been used for successful surface production of invasin from *Yersinia pseudotuberculosis* [30] and the murine chemokine CCL3 [43]. While overproduction of heterologous proteins often severely hampers bacterial growth, the H2-producing strain grew well.

The heterologous prime-boost strategy implies the use of different routes for administration of the same antigen and several studies have demonstrated this strategy to be superior to homologous prime-boost strategies [44]. The strategy is particularly relevant to some of the most challenging vaccines, such as vaccines against HIV-1, tuberculosis and malaria [44]. Heterologous prime-boost vaccination against HIV-1 [45] or *Plasmodium falciparum* [46], in which mucosal priming was followed by systemic immunization via parenteral routes, was successful in pre-clinical experiments. Heterologous prime-boost regimens may induce both systemic and mucosal immunity, which is a desirable trait of a future vaccine against *C*. *trachomatis* [47].

In the present study, we applied a heterologous prime-boost strategy, where systemic immunization was followed by mucosal administration of a booster. We used the subcutaneous route for priming with H2-CAF01, followed by intranasal boosting with *L*. *plantarum* producing H2. Results from the cellular assays showed increased secretion of IFN-γ in both splenocytes (Fig 5A) and PBMC (data not shown) from mice boosted with *Lp*_H2 (group H2/Lp_H2). No detectable induction of cellular responses was observed for the group that had been intranasally immunized with *Lp*_H2 without prior subcutaneous vaccination, suggesting that priming was essential for induction of IFN-γ responses. Similar importance of parenteral priming vaccination has been demonstrated in a tuberculosis study, where priming turned out to be fundamental for eliciting CD4+ T cell response [48].

As expected, mice immunized with a parental vaccine elicited IgG response in blood that did not depend on the boosting with *Lp*_H2, although the H2/Lp_H2 group did show slightly increased IgG levels (Fig 5B). The most important success criteria for our study was the induction of a mucosal immune response, reflected in the generation of H2-specific IgA [28]. Notably, IgA production, both in plasma and at mucosal sites, was only obtained in mice that had been intranasally boosted with *Lp_*H2 (Fig 5C, 5D, 5E, 5F and 5G), and the same trend was observed when we tested recognition of a neutralizing epitope by IgA (S1 Fig). This clearly shows the validity and the potential of the immunization strategy explored here.

The specific IgA response in the vaginal cavity (Fig 5D) is particularly important since such a response could be involved in protection against genital *C*. *trachomatis* infection. Our data, demonstrating successful use of the intranasal route to induce vaginal IgA, are in agreement with previous experiments, also using the intranasal route to generate a vaginal IgA response [28, 49, 50]. Thus, accumulating data show that the intranasal route is suitable for vaccination against genital *C*. *trachomatis* infections. In the more general context of developing *Lactobacillus*-based vaccines, it is important to note that the present results show that the bacterium displaying the *Chlamydia* antigen successfully delivered the antigen to the mucosal immune system and led to generation of antigen-specific IgA. It should be noted, however, that the measured IgA levels were low and that further optimization of this approach thus seems needed. In such further studies, the observed heterogeneity in the immune responses (Fig 5A and 5F) also deserves attention.

To summarize, our results show that *L*. *plantarum* displaying the H2 antigen on its surface was able to induce specific cellular and humoral immune responses, when administered intranasally to mice that had been subcutaneously primed with H2-CAF01. Moreover, the bacterial vaccine elicited H2-specific IgA responses in vaginal mucosa, which is of key importance for controlling *C*. *trachomatis* genital infections. The results of this study encourage further analysis and optimization of *Lactobacillus*-based strategies, to achieve increased IgA titers and to analyze if this strategy in fact induces protective tissue-resident B and T cells in the vagina [51].

## Supporting information

S1 Fig Mucosal IgA responses against the neutralizing epitope of the H2 antigen. Lung samples (A) and vaginal washes (B) from the individual mice were added to plates coated the LNPTIAG epitope-containing P30 peptide or with H2, and levels of bound IgA were measured by ELISA. The results per group are presented as a mean ± SEM (n = 8). Statistical significance was determined using one-way ANOVA with Tukey's post hoc test and is indicated as follows: *, p < 0.05.
(EPS)
